# Inference of neuronal network spike dynamics and topology from calcium imaging data

**DOI:** 10.3389/fncir.2013.00201

**Published:** 2013-12-24

**Authors:** Henry Lütcke, Felipe Gerhard, Friedemann Zenke, Wulfram Gerstner, Fritjof Helmchen

**Affiliations:** ^1^Laboratory of Neural Circuit Dynamics, Brain Research Institute, University of ZurichZurich, Switzerland; ^2^School of Computer and Communication Sciences and School of Life Sciences, Brain-Mind Institute, Ecole Polytechnique Fédérale de LausanneLausanne, Switzerland; ^3^Neuroscience Center Zurich, University of Zurich and ETH ZurichZurich, Switzerland

**Keywords:** calcium, action potential, reconstruction, connectivity, scale-free, hub neurons

## Abstract

Two-photon calcium imaging enables functional analysis of neuronal circuits by inferring action potential (AP) occurrence (“spike trains”) from cellular fluorescence signals. It remains unclear how experimental parameters such as signal-to-noise ratio (SNR) and acquisition rate affect spike inference and whether additional information about network structure can be extracted. Here we present a simulation framework for quantitatively assessing how well spike dynamics and network topology can be inferred from noisy calcium imaging data. For simulated AP-evoked calcium transients in neocortical pyramidal cells, we analyzed the quality of spike inference as a function of SNR and data acquisition rate using a recently introduced peeling algorithm. Given experimentally attainable values of SNR and acquisition rate, neural spike trains could be reconstructed accurately and with up to millisecond precision. We then applied statistical neuronal network models to explore how remaining uncertainties in spike inference affect estimates of network connectivity and topological features of network organization. We define the experimental conditions suitable for inferring whether the network has a scale-free structure and determine how well hub neurons can be identified. Our findings provide a benchmark for future calcium imaging studies that aim to reliably infer neuronal network properties.

## Introduction

Information processing in the nervous system is mediated by distributed spatiotemporal spiking activity patterns in networks of neurons. Experimentally, neuronal network dynamics have been difficult to investigate, especially under the relevant *in vivo* conditions for studying the neural underpinnings of sensory, motor, and cognitive phenomena. While multi-electrode arrays or silicon-based multi-electrode probes allow for simultaneous electrophysiological recording of spike trains from tens to hundreds of neurons with high temporal precision (Buzsaki, 2004), these techniques also suffer from a number of limitations. Assigning the recorded signal to multiple neurons in the proximity of the recording electrode remains challenging (“spike-sorting problem”) (Einevoll et al., 2011) and, most importantly, multi-electrodes sample neural tissue non-homogeneously, with highly active neurons in the vicinity of the recording electrodes being overrepresented (Olshausen and Field, 2005). This sampling bias can lead to spurious results in effective connectivity studies (Gerhard et al., 2011). Finally, extracellular multi-unit recordings commonly provide little information about cell type identity and spatial distribution of the recorded neurons.

Two-photon calcium imaging in the living brain has emerged as a powerful alternative technique, using either synthetic small-molecule or genetically-encoded calcium indicators (reviewed in Garaschuk et al., 2006; Grienberger and Konnerth, 2012; Knopfel, 2012; Looger and Griesbeck, 2012). Calcium signals imaged with high-affinity indicators can serve as proxy of spike dynamics because each action potential (AP) is associated with a rather stereotypical somatic calcium influx causing a characteristic elementary calcium transient. Calcium imaging addresses several of the limitations inherent in multi-electrode recordings. Most importantly, it enables comprehensive sampling of the activity of many, if not all, neurons within a local population, currently up to about 500 neurons with cell number trading off against temporal resolution (1 Hz to 1 kHz) and signal-to-noise ratio (SNR) (Grewe and Helmchen, 2009; Lütcke and Helmchen, 2011). Moreover, calcium signals can be assigned unequivocally to individual neurons, permitting the analysis of the spatial distribution of neuronal activity patterns (Dombeck et al., 2009; Kampa et al., 2011) and long-term repeated functional probing of the exact same neuronal populations (Margolis et al., 2012; Lütcke et al., 2013). Finally, calcium imaging may be combined with genetic tools or *post hoc* labeling approaches to identify specific subtypes of neurons (Kerlin et al., 2010; Hofer et al., 2011; Langer and Helmchen, 2012), or with retrograde tracers to reveal long-range projection patterns of the imaged neurons (Chen et al., 2013a).

Because two-photon imaging conventionally is based on relatively slow frame rates (1–15 Hz), the majority of calcium imaging studies to date have focused on static neuronal properties such as sensory tuning curves (Ohki et al., 2005, 2006; Rothschild et al., 2010). In recent years, however, advanced laser scanning methods have been developed that enable high-speed population imaging (25 Hz and higher, up to 1 kHz) (Nikolenko et al., 2008; Otsu et al., 2008; Grewe et al., 2010; Ranganathan and Koester, 2010; Bonin et al., 2011; Katona et al., 2012). In some cases spike times could be inferred with near-millisecond temporal precision (Grewe et al., 2010; Ranganathan and Koester, 2010; Fernández-Alfonso et al., 2013). In combination with dedicated analysis routines, high-speed two-photon calcium imaging should thus be capable, in principle, to report dynamic AP patterns in local neuronal populations. Besides providing unique opportunities to measure network activity *in vivo*, such experiments could even make it possible to extract structural information about network connectivity and topology, given sufficient accuracy of spike inference in the network.

A plethora of different algorithms have been developed to infer the spike train underlying a particular observed calcium indicator fluorescence trace. They can be broadly classified into deconvolution-based approaches to estimate changes in neuronal activity without attempting to reconstruct the occurrence of individual spikes (Yaksi and Friedrich, 2006; Vogelstein et al., 2009, 2010), template-matching techniques that infer spike times based on knowledge of the prototypical waveform of the single-AP evoked calcium transient (Kerr et al., 2007; Greenberg et al., 2008; Grewe et al., 2010; Onativia et al., 2013) and machine-learning algorithms, which require training on data sets with known “ground truth” (Sasaki et al., 2008). Little attention has been given, however, to a systematic study of how spike inference is influenced by different experimental parameters, such as SNR or signal acquisition rate. Experimentally, this is difficult to address because it would require a whole set of experiments with simultaneous *in vivo* calcium imaging and electrophysiological recordings from many neurons under various conditions. At present, only selective calibration experiments are feasible, testing the sensitivity of calcium indicators by simultaneous imaging and recording of single neurons (Kerr et al., 2005). Nonetheless, a thorough investigation of the effect of experimental parameters on spike inference would be an invaluable resource for experimentalists in order to plan experiments as well as interpret results, especially in view of the recent developments in imaging technology and indicator design. Only a few studies, employing mostly theoretical analysis or numerical simulations, have started to more systematically analyze the prospects and limits of spike inference from optical recordings (Sjulson and Miesenbock, 2007; Wilt et al., 2013) as well as of extracting network information from inferred population spike dynamics (Vogelstein et al., 2010; Mishchenko et al., 2011; Stetter et al., 2012).

Here, we present a quantitative simulation framework to generate two-photon calcium imaging signals from the spiking activity of neocortical neurons, simulated either for individual cells or for subsets of neurons within a large-scale network. Using simulated single-neuron fluorescence signals we first characterize the influence of relevant parameters of the reconstruction algorithm on spike inference, exemplified here for the recently introduced “peeling” algorithm that iteratively removes detected single-AP evoked calcium transients from the observed fluorescence signal (Grewe et al., 2010). To guide experimentalists, we provide a systematic and quantitative analysis of the impact of several parameters related either to imaging data acquisition, indicator properties, or inference routine. We evaluate how these parameters—within value ranges relevant for real experiments—influence the fractions of correctly inferred and falsely discovered APs as well as the temporal precision of spike inference. We also extend the peeling algorithm to consider calcium indicator saturation at high spike rates.

Using a large-scale neuronal network simulation, we subsequently show that structural connectivity between neurons can be partially inferred even from limited amounts of imaging data, from a sparse subset of the population, and under fluctuating, unobserved, common input. We show that parametric statistical models can extract substantially more information than pairwise cross-correlational analysis. Based on our spike inference analysis we then examine to what extent the inference of network properties is expected to deteriorate for realistic calcium imaging conditions. Finally, we investigate whether statistical network properties, such as scale-free topologies (Barabasi and Albert, 1999) and hub neurons (Feldt et al., 2011) can be recovered from the estimated connectivity matrices. Our results suggest that the current state-of-the-art in calcium imaging technology not only comes very close to the criteria required for reliable and accurate spike inference in neuronal networks but also enables at least in part to gain additional information about network connectivity and topological features.

## Results

### A framework for simulations of neural network calcium imaging data

**Figure 1:**
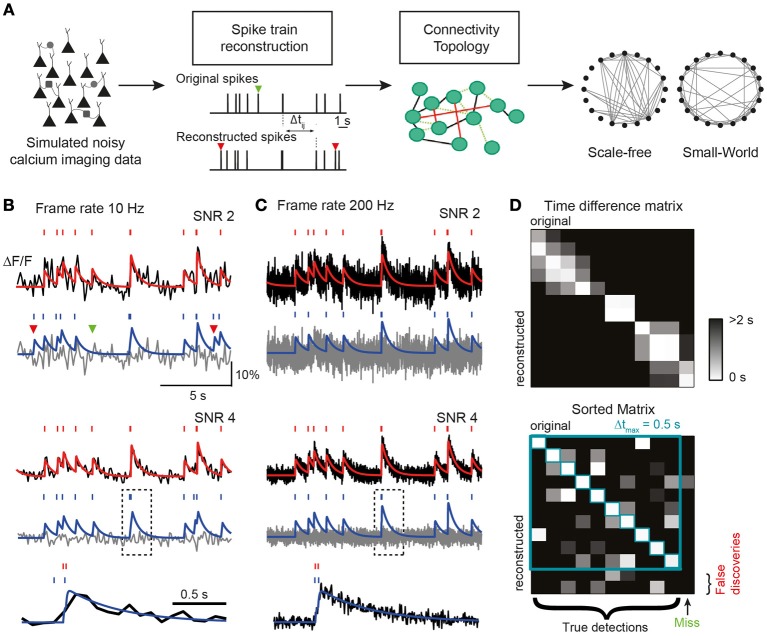
**(A)** Conceptual link between neuronal network dynamics and structure in our study. Network dynamics measurable with calcium imaging techniques is simulated to investigate how well spike trains can be reconstructed for a broad value range of the most important experimental parameters. Reconstruction performance is condensed in three key parameters (TPR, true positive rate; FDR, false discovery rate; and σ_Δ*t*_, temporal precision), which are used to analyze how well structural network properties such as topological characteristics (right) can be revealed depending on the attainable accuracy of spike train reconstruction. Missed (green) and falsely detected (red) spikes indicated by arrowheads. **(B–D)** Simulation of calcium traces from spike trains and subsequent reconstruction of firing pattern. **(B)** Top: simulated noise-free (red) and noisy (black) calcium traces for the example Poisson spike train (*SNR* = 2; *f* = 10 Hz). Bottom: reconstruction of spike train from simulated noisy calcium trace using the peeling algorithm. Reconstructed spike train and model calcium trace in blue. Gray: residual calcium trace. Note the missed (green) and two false spikes (red). Middle: same traces but with a better SNR of 4. Bottom: expanded view of example calcium transient in gray box on faster time scale. Note the timing imprecision of reconstructed spike timing due to the low frame rate. **(C)** Same data as in **(B)** but with a higher frame rate. Note the improved reconstruction, especially at the faster time scale (bottom). **(D)** Illustration of simulated original spike train and reconstructed spike train (*SNR* = 2; *f* = 10 Hz). Spikes are matched based on the Δ*t* matrix of all spike-pair-intervals Δ*t*_*ij*_ between original and reconstructed trains. After sorting the matrix according to the rank of Δ*t* and applying a threshold Δ*t*_max_, spikes are either matched or remain as “misses” or “false discoveries.” Unmatched spikes *i* with Δ*t*_*ij*_ > Δ*t*_max_ for all *j* are undetected original spikes (misses) and unmatched spikes *j* with Δ*t*_*ij*_ > Δ*t*_max_ for all *i* are spurious reconstructed spikes (false discoveries).

Our first aim was to create a simulation environment for mimicking actually recorded calcium indicator fluorescence traces (both in single neurons and in a network of spiking neurons), which then can be treated in exactly the same way as real experimental data. The advantages of exploring simulated fluorescence transients are: (1) the reconstructed spike trains can always be compared to the ground truth of original spike trains; (2) many artificial spike trains can be easily generated; and (3) spike trains in networks with known connectivity can be utilized to explore the possibility of extracting information about network structure from calcium imaging data. As a result, different parameters related either to the network itself, the experimental conditions or the analysis routines can be systematically varied to evaluate their relative influences (Figure 1A).

To generate artificial AP-evoked fluorescence signals, we simulated spike trains either in single neurons (Poisson process with time-independent mean firing rate r) or in a simulated network of leaky integrate-and-fire neurons (see Materials and Methods). Spike trains were converted to fluorescence signals, taking into account their relationship to changes in intracellular free calcium concentration ([Ca^2+^]_*i*_). As commonly done for experimental data, we expressed calcium signals as relative percentage fluorescence changes (Δ*F*/*F*). We first presumed a linear relationship between [Ca^2+^]_*i*_ changes and Δ*F*/*F*, which is justified for relatively isolated brief transients as they occur for sparse spiking (for treatment of non-linear indicator saturation at high firing rates see below). This is self-explanatory. In the linear case, Δ*F*/*F* traces were generated by convolving spike trains with a kernel with a fast exponential rise (time constant τ_on_) and a slower exponential decay (time constant τ_off_), mimicking the stereotyped single AP-evoked calcium transient typically observed in neocortical pyramidal neurons with the synthetic indicator Oregon Green BAPTA-1 (OGB-1) (Kerr et al., 2005; Grewe et al., 2010). Realistic Gaussian noise was added to the simulated calcium signals to yield different SNRs (see Materials and Methods). SNR was defined as the ratio of the peak amplitude of the elementary calcium transient divided by the standard deviation of baseline activity. Finally, noisy calcium traces were subsampled from the original temporal resolution (2 kHz) to a given target frame rate, *f*, by selecting the center data point of each time interval (1/*f*). This procedure resembles the laser scanning approach used in most two-photon microscopes. Figures 1B,C show examples of simulated Δ*F*/*F* traces for two different frame rates (10 and 200 Hz) and noise levels (SNR 2 and 4).

In the following, we address three major questions with this simulation framework. First, how good is the reconstruction of spike trains in individual neurons under systematically varied conditions? Second, in how far can one extract information about physiological connectivity from the more or less accurate inferred spike times in the network? Finally, what level of reconstruction performance is necessary to infer statistical features of the underlying network topology, such as the identification of hub neurons or scale-free properties?

### Analysis of spike inference from simulated calcium signals in individual neurons

Our simulation framework provides a convenient strategy for the comprehensive evaluation of various algorithms that have been devised for inferring spike trains from noisy calcium recordings (Greenberg et al., 2008; Sasaki et al., 2008; Grewe et al., 2010; Vogelstein et al., 2010). However, a comparison of different algorithms was not the goal of this study. Rather, we exemplify how the performance of one particular spike reconstruction approach, the peeling algorithm introduced in (Grewe et al., 2010), depends on different experimental parameters. In principle the same systematic approach can be followed for other reconstruction algorithms using the MATLAB code provided (Supplementary Materials).

The peeling algorithm is based on iterative subtraction of a template elementary calcium transient at event onset times detected by a Schmitt trigger routine, thus “peeling” away calcium transients until a residual noise trace remains (Figures 1B–D). Besides the parameters describing the template calcium transient, the main parameters of the peeling algorithm are two thresholds (an initial high-passing and a second low-passing one) and the minimum duration between the two threshold crossings that has to pass by in order to count as an event (see Materials and Methods). The peeling algorithm returns a list of spike times in continuous time (independent of acquisition rate). Examples of spike trains reconstructed from simulated calcium traces using the peeling algorithm are shown in Figures 1B,C. An advantage of the peeling algorithm is that it can be extended to conditions, under which indicator saturation becomes relevant (see below).

The reconstructed spike train may contain false negatives (missed spikes) and false positives (falsely discovered spikes). To quantify the performance of the inference algorithm, we compared the original and reconstructed spike train as follows. We first calculated a matrix of spike time differences (Δ*t*) for all pairs of original and reconstructed spikes (Figure 1D). We then assigned the first spike pair based on the smallest time difference, repeated this best-matching approach for the remaining spikes, and iterated until no further pair was found to meet a tolerance time window criterion (by default Δ*t*_max_ = 0.5 s). The remaining “lonely” spikes constituted missed and falsely discovered spikes, respectively (Figure 1D). The outcome of this comparative approach was condensed in two main parameters: the true positive rate TPR_AP_ (number of correctly detected spikes divided by the original number of spikes; also called “sensitivity” or “recall”) and the false discovery rate FDR_AP_ (number of falsely discovered spikes divided by the number of reconstructed spikes, with (1-FDR_AP_) also referred to as “precision”). We preferred to use the FDR rather than the false positive rate (FPR, number of time bins with falsely reconstructed spikes divided by the total number of time bins without original spike) because FPR depends on time binning and becomes arbitrarily small for high acquisition rate and sparse spiking. TPR_AP_ and FDR_AP_ on the other hand provide an intuitive quantification of the fractions of accurately and inaccurately detected spikes (see also Materials and Methods). We furthermore quantified the temporal precision of correctly retrieved spikes as the mean and standard deviation (meanΔ*t* ± σ_Δ*t*_) of the difference between matched reconstructed and original spike times.

**Figure 2:**
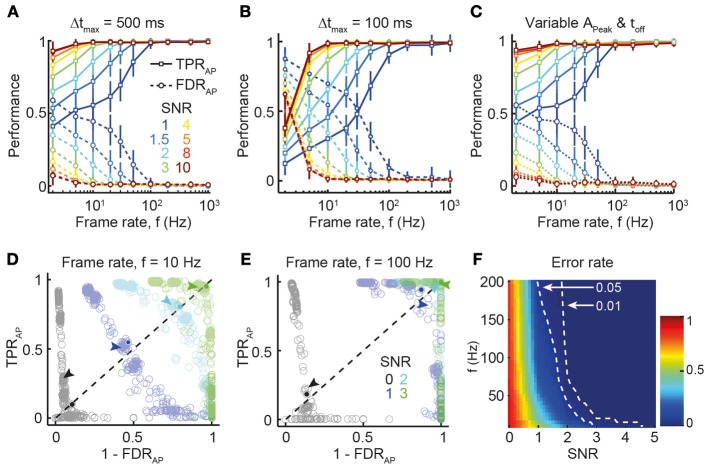
**Dependence of spike reconstruction performance on SNR and frame rate. (A)** TPR_AP_ (solid lines) and FDR_AP_ (dotted lines) as function of frame rate (x-axis) and SNR (different colors). Temporal window for declaring correct AP detection: 500 ms. Mean ± SD. **(B)** Same analysis as **(A)** but with narrower temporal window for declaring correct AP detection (100 ms). **(C)** Similar simulation as in **(A,B)**, but with variable calcium transient parameters *A*_Peak_ and τ_off_. For each AP, corresponding values for *A*_Peak_ and τ_off_ were selected from a normal distribution with mean *A*_Peak_ = 7% and mean τ_off_ = 1 s and *SD* = 10% of the mean value. Temporal window for declaring correct AP detection: 500 ms. **(D)** PR-curve showing the trade-off between TPR_AP_ and FDR_AP_ for different SNR at *f* = 10 Hz (see SNR legend in **E**). Data points indicate different combinations of Schmitt-trigger thresholds for the spike reconstruction algorithm (see Materials and Methods). Arrows mark the data points corresponding to thresholds employed for reconstruction in this study. Solid circles indicate the break-even point used for quantifying overall reconstruction performance. The error rate is the normalized distance of the break-even point from the top-right corner (perfect reconstruction accuracy). **(E)** Same analysis as **(D)** at *f* = 100 Hz frame rate. Marked data points for SNR 2 and 3 are superimposed in top-right corner, indicating near-perfect reconstruction accuracy. **(F)** Error rate for all combinations of SNR and frame rate (linear interpolation between simulated parameter combinations). Dashed lines indicated thresholds for 0.01 and 0.05 error rates.

We first investigated how SNR and acquisition rate influence spike inference under conditions commonly observed for cortical pyramidal neurons (assuming OGB-1 labeling: single-AP peak Δ*F*/*F* amplitude 7%; decay time constant 1 s; low average firing rate of 0.2 Hz). Figure 2A summarizes the reconstruction accuracy in terms of TPR_AP_ and FDR_AP_ for different SNR levels and frame rates. As expected, lower noise levels and faster frame rates were associated with better reconstruction performance. Near-perfect reconstruction accuracy was achieved at surprisingly low frame rates, with little improvement above 30 Hz (Figure 2A), in agreement with the experimentally verified performance of the peeling algorithm *TPR*_AP_ = 95.5% and *FDR*_AP_ = 1.5% for 181–490 Hz acquisition rate and SNR levels between 2 and 5 (Grewe et al., 2010). Even at very high noise levels (SNR 1–1.5) of simulated traces, very good reconstruction performance was attained at higher sampling rates (≥100 Hz). Note that choosing a shorter temporal window for declaring correct AP detections (Δ*t*_max_ = 0.1 s instead of 0.5 s) impaired reconstruction accuracy at lower frame rates (≤10 Hz) whereas accuracy at high frame rates remained largely unaffected (Figure 2B). We next asked how spike inference might be influenced by additional noise factors, such as variability in the main parameters describing the single AP-evoked calcium transient (*A*_Peak_ and τ_off_). To address this question, we selected variable values of *A*_Peak_ and τ_off_ for the calcium transient model of each AP, based on a normal distribution with mean *A*_Peak_ = 7% and mean τ_off_ = 1 s and *SD* = 10% of the mean value (i.e., 0.7% for A_Peak_ and 0.1 s for τ_off_). Results of this simulation (Figure 2C) revealed only minor decreases in reconstruction performance, compared to the noiseless parameter scenario (Figure 2A), demonstrating that spike inference with the peeling algorithm is relatively robust against small, random fluctuations in critical parameters.

Spike reconstruction performance is not only determined by imaging parameters, such as SNR or acquisition rate, but also by properties of the detection algorithm itself, notably the settings for different thresholds (see Materials and Methods). To evaluate the effects of different decision criteria (thresholds) on signal detection, we investigated the trade-off between falsely discovered spikes (1 − FDR_AP_; “precision”) and correctly detected spikes (TPR_AP_; “recall”) [so-called “precision-recall” (PR) curve; Figures 2D,E]. Using error rate α_AP_ (see Materials and Methods) as performance metric, we confirmed that sensitive spike detection is achievable at relatively low SNR, provided that frame rates are high enough (e.g., >100 Hz; Figure 2F).

### Influence of indicator properties and parameter choices

**Figure 3:**
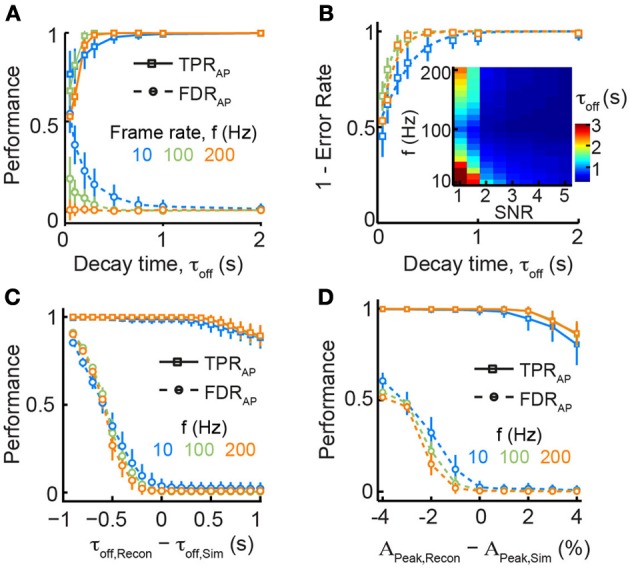
**Dependence of spike train reconstruction on assumed calcium transient parameters. (A)** TPR_AP_ (solid lines) and FDR_AP_ (dotted lines) as function of decay time τ_Off_ and frame rate *f* for fixed *SNR* = 3. Note that faster decay times lead to markedly reduced reconstruction performance especially at slow sampling rates. **(B)** Overall performance accuracy (1 − α_AP_) increases for longer decay times. Dashed lines are logistic fits to the data points. The fits were used to compute the minimal decay time that would be necessary to reach an error rate α_AP_ = 0.05 (inset). **(C)** Robustness of reconstruction performance against deviations from model parameters: decay time, τ_Off_. Data were simulated with τ_Off, Sim_ = 1 s and reconstruction was performed assuming different values for τ_Off, Recon_. Note that reconstruction with faster decay times leads to a strong increase in FDR_AP_ whereas slower decay times lead to a more graceful deterioration of TPR_AP_ (*SNR* = 3). **(D)** Robustness of reconstruction performance against deviations from model parameters: peak calcium amplitude, *A*_Peak_. Data were simulated with *A*_Peak, Sim_ = 7% ΔF/F and reconstruction was performed assuming different values for *A*_Peak, Recon_. Reconstruction with smaller *A*_Peak_ results in many more falsely detected APs whereas over-estimation of *A*_Peak_ leads to a more graceful deterioration of TPR_AP_ (*SNR* = 3).

We next investigated how properties of the calcium indicator itself might affect spike reconstruction performance. Calcium indicators are Ca^2+^-binding molecules with characteristic binding kinetics and affinity, and they can be applied in different concentrations. These parameters affect the shape of recorded fluorescence transients, in particular the onset time, the peak fluorescence (and thus SNR), and the decay time course (Göbel and Helmchen, 2007). For the majority of commonly used calcium indicators these properties have been measured and they are usually reported for new indicators (see for example We asked how variation in one of the most important parameters governed by indicator properties, the decay time constant τ_off_, affects reconstruction performance given different experimental constraints (notably frame rate and SNR). For common synthetic indicators such as OGB-1, τ_off_ is about 0.5–1 s for typical indicator concentrations, while other recently developed highly sensitive genetically encoded calcium indicators (GECIs) can display slower decays (e.g., τ_off_ = 2–4 s for YC-Nano15) (Horikawa et al., 2010). Our simulations clearly show that longer decay times do not preclude accurate detection of APs, at least for sparse firing regimes (Figure 3A). Contrarily, we observed a rapid deterioration of reconstruction performance for faster decay times (τ_off_ < 0.5 s), most notably at lower acquisition rates and SNRs (Figure 3B). Intuitively, this can be explained by a too low sampling density resulting in frequent misses of the brief initial peak of calcium transients. These results suggest that the use of new calcium indicators with faster fluorescence decay times (Chen et al., 2013b) would only be beneficial in combination with imaging at sufficiently high speed.

Algorithms for spike inference from fluorescence signals typically rely on the use of a standard single-AP calcium transient (see above). Although the shape of this elementary calcium transient is rather stereotyped (as long as saturation can be neglected; see below) and often well-characterized for a certain cell type, variations among individual cells do exist and uncertainties remain about the values to choose for the parameters of the reconstruction algorithm. We therefore explored to what extent reconstruction performance depends on the accurate choice of parameters for the elementary calcium transient. We simulated AP-evoked Δ*F*/*F* traces with a fixed decay time constant τ_off, Sim_ of 1 s but for spike train reconstruction we systematically varied the presumed decay time constant τ_off, Recon_ between 0.1 and 2 s (Figure 3C). Reconstruction with shorter τ_off, Recon_ dramatically increased the fraction of falsely detected APs while TPR_AP_ remained unaltered. Choosing too long decay times for reconstruction led to a smaller decline of TPR_AP_ while FDR_AP_ remained low. Overall spike reconstruction accuracy decreased by 35% for a 50% decrease in assumed τ_off, Recon_ (τ_off, Recon_ = 0.5 s) while a corresponding doubling (τ_off, Recon_ = 2 s) reduced accuracy by only 10% (Figure 3C). Another parameter that may be partly unknown under experimental conditions is the peak fluorescence *A*_Peak_ for a single AP. We found that under-estimation of *A*_Peak_ led to a dramatic increase in FDR_AP_ while over-estimation again caused a more graceful degradation in TPR_AP_ (Figure 3D). Given that the true values for *A*_Peak_ and τ_off_ are frequently unknown under experimental conditions, our analysis suggests that over-estimating these parameter may be a good strategy to optimize spike reconstruction accuracy (Figures 3C,D).

### Spike inference under conditions of indicator saturation

**Figure 4:**
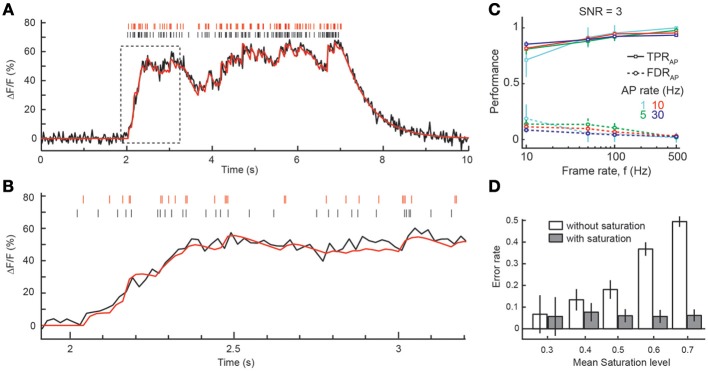
**Spike inference from episodes with high firing rates. (A)** Example simulation of an episode of 30 Hz firing for 5 s. The fluorescence trace was simulated with a frame rate of 50 Hz and *SNR* = 3 using the model of calcium dynamics including indicator saturation. Black: simulated APs and Δ*F*/*F* trace. Red: reconstructed APs and result of peeling algorithm with saturation model. **(B)** Zoom of initial part of the episode (boxed region in **A**). Note the decreasing amplitude of single-AP transients at high Δ*F*/*F* due to saturation. **(C)** TPR_AP_ (solid lines) and FDR_AP_ (dotted lines) as function of frame rate (x-axis) and firing rate (different colors). *SNR* = 3. Temporal window for declaring correct AP detection: 100 ms. **(D)** Dependence of error rate on mean saturation level during burst episodes with (gray) and without (white) taking saturation into account in the spike reconstruction with the peeling algorithm. Note the large increase in error rate at high saturation levels when a non-saturating, linear superposition of Δ*F*/*F* transients is wrongly presumed. Analysis in **(D)** is based on simulations with frame rate ≥50 Hz and *SNR* = 3 and 10. All simulated Δ*F*/*F* traces were generated using the model of calcium dynamics including indicator saturation. All panels show mean ± SD.

So far, we have assumed sparse spiking conditions (low firing rate), for which Δ*F*/*F* can be presumed to relate linearly to [Ca^2+^]_*i*_. However, episodes of AP bursts with higher firing rates, e.g., during optimal sensory stimulation (Decharms et al., 1998) or under awake conditions (Greenberg et al., 2008), will cause larger [Ca^2+^]_*i*_ elevations and increasingly drive high-affinity indicators into saturation. We therefore also incorporated saturation effects in our simulation framework and extended the peeling algorithm to account for saturating fluorescence transients during burst episodes of APs (see Materials and Methods). To evaluate the performance of the reconstruction algorithm for higher firing rates, we simulated saturating fluorescence traces in response to 5–10 s long episodes of AP firing rate at 1–30 Hz. At high firing rates fluorescence traces reached Δ*F*/*F* values greater than 60% (Figure 4A), corresponding to saturation levels around 0.7. Due to saturation, the amplitude of individual AP-evoked Δ*F*/*F* transients decreases at elevated [Ca^2+^]_*i*_ levels (Figure 4B). However, taking this saturation effect into account in an improved version of the peeling algorithm (see Materials and Methods) we could still recover the majority of spikes, even at high firing rates, as exemplified for *SNR* = 3 in Figure 4C. When the mean saturation level during the spiking episode increased at higher firing rates, we observed very little, if any, decrease in the overall reconstruction performance. Note, however that this result depended critically on saturation being implemented in the reconstruction step. When we simulated saturating [Ca^2+^]_*i*_ traces but attempted to reconstruct them without taking saturation into account (using the linear approximation), error rates increased dramatically (Figure 4D). In summary, the results of the previous sections demonstrate that the peeling algorithm enables robust and highly accurate spike inference over a large range of imaging, indicator and experimental parameters.

### Temporal precision of spike inference

**Figure 5:**
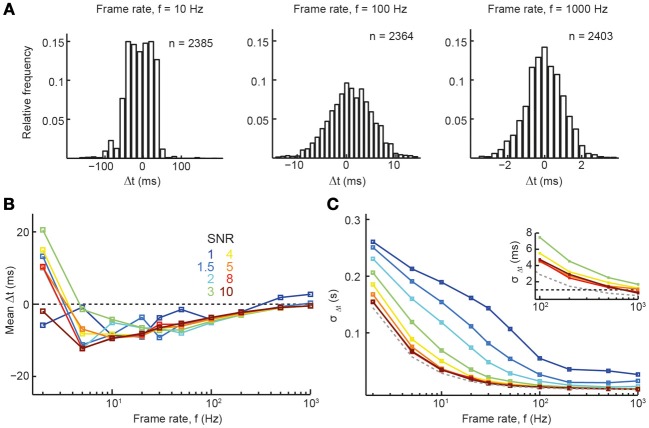
**Precision of spike time inference. (A)** Histogram of the spike time differences between original and reconstructed spike trains for 3 different frame rates (*SNR* = 5). **(B,C)** Summary parameters for the distribution of spike time differences between original and reconstructed spikes. **(B)** Mean spike time difference, Δ*t*. **(C)** Standard deviation of the distribution of spike time differences, σ_Δ*t*_. Dashed gray line: theoretical limit set by frame rate. Dashed black line (inset) indicates 1 ms precision. Relevant simulation parameters: firing rate = 0.2 Hz, A = 7%, τ_On_ = 10 ms, τ_Off_ = 1 s.

Given that accurate spike reconstruction can be achieved with our reconstruction algorithm for a wide range of parameter combinations, we next wanted to explore the temporal precision of accurate reconstructions (returning to low average firing rate of 0.2 Hz). Figure 5A shows example distributions of spike time differences at three different sampling rates (meanΔ*t* ± σ_Δ*t*_ for 10 Hz: −9 ± 35 ms; 100 Hz: −4 ± 5 ms; 1000 Hz: 0 ± 1 ms). Again, these results are in line with the experimentally determined spike time reconstruction precision of the peeling algorithm (SNR 2–5; Grewe et al., 2010). At very high sampling rates and good SNR, spike train reconstruction approached sub-millisecond accuracy, as quantified by the standard deviation (SD) of the Δ*t* distribution (Figures 5B,C; τ_off_ = 1 s, τ_on_ = 10 ms). For example, at a rate of 1 kHz, σ_Δ*t*_ was 0.67 and 0.56 ms for SNR 8 and 10, respectively (Figure 5C, inset), which is close to the limit set by the sampling interval. Of note, temporal precision was little influenced by τ_off_ (data not shown) and only slightly reduced by slower onset times (at τ_on_ = 20 ms, σ_Δ*t*_ was 0.95 and 0.76 ms for SNR 8 and 10, respectively; at τ_on_ = 50 ms, σ_Δ*t*_ was 2.90 and 3.12 ms for SNR 8 and 10, respectively). These results demonstrate that recently developed high-speed imaging approaches should be adequate for accurate spike reconstruction with high temporal precision. Our analysis furthermore suggests that the recent development of high-SNR GECIs (Horikawa et al., 2010; Chen et al., 2013b), even if these exhibit slightly slower onsets, may soon allow for accurate spike reconstruction from calcium imaging data with millisecond precision.

The two variables α_AP_ and σ_Δ*t*_ (ignoring mean Δ*t* which will cancel out in a network reconstruction analysis based on relative differences of spike times of multiple neurons) describe in condensed form the overall performance of spike reconstruction in terms of accuracy and temporal precision. In the second part of our study, our goal was to apply this framework to simulated large-scale networks of spiking neurons with known connectivity and investigate how the attainable levels of spike reconstruction accuracy and temporal precision affect the extraction of synaptic coupling structure between neurons in order to estimate structural network connections within local neuronal populations.

### Simulation of large-scale network dynamics of spiking neurons

**Figure 6:**
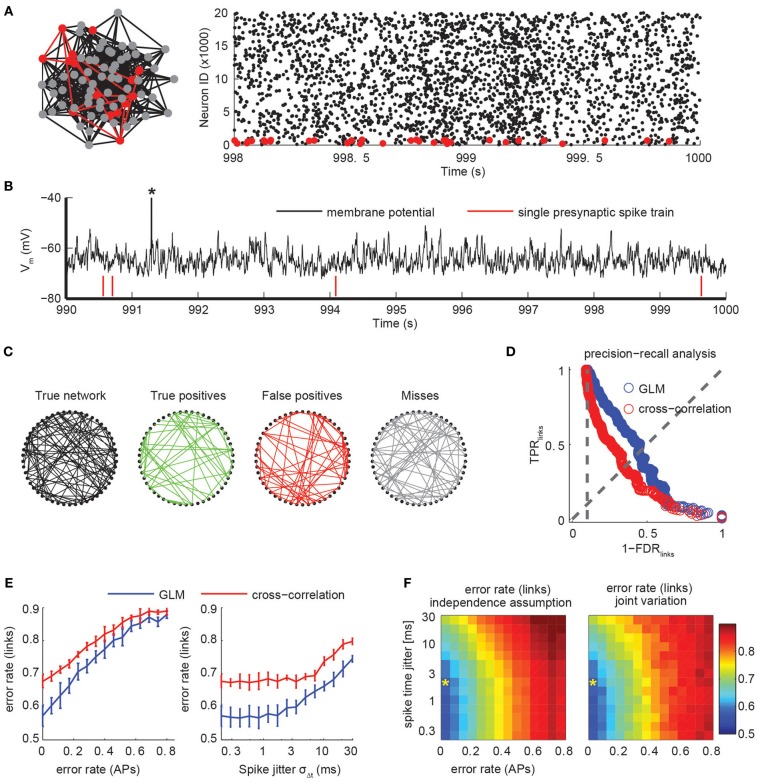
**Connectivity extraction using imperfect spike trains. (A)** A population of 25,000 excitatory and inhibitory neurons with sparse, random connectivity was simulated using integrate-and-fire models with conductance-based synapses. Firing activity was sparse and irregular. Subsets of 50 neurons (red) were randomly selected and only those spike trains were used to infer their mutual synaptic connectivity (left). Raster plot of the activity of the excitatory subpopulation for 2 s (right). Only every third spike is shown. **(B)** Membrane potential of a randomly selected neuron. The average neuron fired sparsely (note truncated AP marked by asterisk). Synaptic couplings were weak so that single presynaptic APs (red ticks) did not elicit APs by itself. **(C)** True network connectivity and connectivity estimated using GLMs for a randomly selected subset of 50 neurons (links divided into correctly identified links in green, false positive links in red and missed links in gray). Error rate was 0.5. **(D)** Trade-off in network reconstruction performance between TPR_links_ and FDR_links_. Performance for unperturbed spike trains using a GLM-based reconstruction (blue) or pairwise cross-correlation analysis (red). Error rate is defined as the intersection with the diagonal and is lower for the GLM than for the cross-correlation for all settings of the threshold. Chance level is indicated with the vertical, dotted line. **(E)** Error rates of the link reconstruction after spike perturbations for variations of the error rate in AP detection only (left, assuming no temporal jitter) and for variation of temporal precision of spike times by introducing a Gaussian jitter with width σ_Δ*t*_ (right; assuming zero error rate in AP reconstruction). Chance level for link reconstruction is 0.9 error rate. Error bars indicate SD over repetitions using random subsets of the network. **(F)** Error rate of link reconstruction as a joint function of error rate (APs) and spike jitter, assuming both effects act independently on the error rate (left) or when jointly varied (right). The similarity indicates that effects of AP detection and its temporal precision act multiplicatively on the expected error rate for link reconstruction. Asterisk indicates the performance level that is realistically achievable with state-of-the-art high speed two-photon calcium imaging (Grewe et al., 2010; Ranganathan and Koester, 2010).

To investigate how the different experimental parameters impact the analysis of the network dynamics, we extended our simulation framework to generate realistic dynamics of cortical networks under *in vivo* recording conditions. We performed large-scale neural network simulations of 25,000 neurons with sparse, random connectivity of 10% and balanced excitatory and inhibitory subpopulations (Figures 6A,B) in line with classical models of cortical networks (Brunel, 2000; Vogels et al., 2005). Neuronal dynamics were modeled with leaky-integrate-and-fire (LIF) models and conductance-based synapses with individual dynamics for GABA, AMPA, and NMDA conductances (see Materials and Methods). Despite their phenomenological nature, LIF models have shown good to excellent correspondence with the complex response properties of single neurons (Badel et al., 2008; Mensi et al., 2012). In addition to the synaptic input from within the simulated population, each neuron received additional correlated Poisson spike trains whose rates were temporally modulated on all possible time scales to mimic shared input from other cortical layers and areas.

The resulting network state was balanced with irregular, asynchronous activity with sparse average firing rates of around 0.2 Hz and global rate fluctuations (see raster plot in Figure 6A). The explicit modeling of slow NMDA conductances (time scale τ^NMDA^=100 ms) led to a more decorrelated activity than in standard neural network simulations (not shown). Because of the size of the simulated network, individual synaptic couplings were relatively weak and single presynaptic APs elicited small postsynaptic potentials and did typically not trigger postsynaptic APs (Figure 6B).

How much can we learn about the structure and topology of the network by observing its activity and dynamics through functional signals as provided by calcium imaging experiments? Incomplete and finite observations as well as intrinsic noise sources fundamentally limit the ability to infer structural connectivity from functional signals. Experimentally, only a small fraction of all interacting circuit elements can be recorded for a limited amount of time. We respected this limitation in our simulation framework by randomly picking 50 (excitatory) neurons from the whole population and extracting their coupling structure despite the fact that there were 24,950 unobserved neurons in addition to correlated, fluctuating, external noise sources (Figures 6A,B). Furthermore, we did not allow arbitrarily long recording sessions, but limited ourselves to what could be observed with less than 3 h of simulated, sparse activity. These numbers were chosen to be in the realm of currently achievable experimental conditions for current high-speed calcium imaging set-ups.

### Inference of structural connectivity from network activity: establishing an upper limit

With the aforementioned limitations, we do not expect to unambiguously recover the network connectivity from observing network activity *even if* we had access to the unperturbed spiking activity with full temporal resolution. We therefore proceeded by first establishing an estimate of the upper bound of how well the network structure could be reconstructed in the case of infinitely precise spike time measurements. Subsequently, we analyzed how the SNR and sampling rate of calcium imaging experiments influence the reconstruction performance relative to this reference value.

To extract the coupling structure from the spike sequence of the subset of 50 neurons we used non-linear point process models (Generalized Linear Models, GLM; see Materials and Methods), which recently have been applied on electrophysiological data (Pillow et al., 2008; Vidne et al., 2012). Briefly, a probabilistic model is fit for each neuron that explains the observed spike times using the neuron's previous activity and a constant baseline rate. Causal couplings between neurons are introduced through parameterized kernels that describe how spikes of putatively presynaptic neurons modulate the spiking probability of the modeled neuron. The model is considerably simpler than the neuron models used in the simulation, e.g., the model is unaware of the true simulation parameters and timescales and does not explicitly model the shared input from the external Poisson processes, making connectivity extraction a non-trivial task. Features of the estimated coupling filters, such as their size or statistical significance, can be used to assign a single coupling strength for each possible directed connection (Gerhard et al., 2011, 2013). After applying a threshold, we obtain an estimate of the binary connectivity structure of the network. For a given threshold, the performance of the reconstruction algorithm can be summarized in the fraction of correctly identified couplings, TPR_links_, and the fraction of erroneously inferred connections among all detected links, FDR_links_ (Figure 6C). Analogous to the quantification of spike train reconstruction performance, the trade-off between the two quantities is given by the choice of threshold as visualized in a PR-curve (Figure 6D). In the following, we summarize the threshold-independent performance of a network reconstruction using the same measure (as in the first part on spike train inference) by the achieved error rate α_links_.

As a result, we found that with a simulated recording time of less than 3 h (or ~2000 observed spikes per neuron), we were able to achieve an error rate between 0.5 and 0.6 (Figure 6D). This provides an optimal lower bound on the achievable error rate under conditions of a noiseless spike detector. We note that a naive analysis based on pairwise spike train cross-correlations resulted in significantly fewer correct links and more false positives (error rates close to 0.7, Figure 6D), indicating the necessity of using modern statistical models to infer network structure from functional imaging.

### Connectivity inference after imperfect spike train reconstruction

An advantage of the systematic approach followed for the spike train reconstruction from noisy calcium signals is that once the key parameters α_AP_ and σ_Δ*t*_ have been determined, we can apply a surrogate transformation to the simulated spike trains of the network simulation and investigate to what degree the quality of spike train reconstruction impacts our ability to draw conclusions about the network connectivity relative to the upper bound established above. We repeated the connectivity reconstruction procedure as described above for perturbed spike trains (see Materials and Methods) and evaluated how the different performance metrics affect the connectivity inference.

First, we looked at the impact of the two parameters independently (Figure 6E). As expected, the measured error rate in the link reconstruction increased with increasing error rate in the AP reconstruction (Figure 6E, left). Given the broad range of frame rates and SNR in calcium imaging for which low error rates α_AP_ were achieved (Figure 2), error rates α_links_ close to the best value (~0.55) should be reachable for many experimental conditions. With spike trains perturbed by introducing a temporal jitter σ_Δ*t*_, error rates in the link reconstruction increase in a more graceful manner, reaching chance level only for σ_Δ*t*_ > 30 ms (Figure 6E, right). At experimentally attainable temporal precision (σ_Δ*t*_ = 1–3 ms; see above), error rates are indistinguishable from the perfect recovery case. For comparison, we also indicated the performance reachable using a standard pairwise cross-correlational analysis, which produced substantially higher error rates than the point process models for all relevant parameter regimes (Figure 6E).

We then jointly varied α_AP_ and σ_Δ*t*_ and found that the error rate in the link reconstruction can be well-predicted by the assumption that contributions from the two factors act independently and in a multiplicative way on α_links_ (Figure 6F).

In summary, our analysis indicates that GLMs allow at least partial extraction of effective network connectivity from imperfectly reconstructed spike trains of moderate length from spontaneous, asynchronous network activity. Importantly, we show that a temporal precision of spike reconstruction in the millisecond-range is the major determinant for accurate estimation of neuronal couplings, given that near-perfect reconstruction accuracy can be achieved under a wide range of imaging conditions (α_AP_ ~ 0, see above). This finding further highlights the importance of recently developed high-speed imaging approaches.

### Identification of graph topology

Through our simulation framework, we estimate that noisy calcium signals from a set of neurons immersed in a larger cortical neural population contain significant information about the underlying network structure that goes beyond what can be trivially extracted using cross-correlational analysis. Absolute reconstruction performance is, however, currently limited by several factors, some of which are at least partially under the control of the experimentalist. We therefore asked what level of reconstruction will be sufficient for inferring high-level features and statistical properties of the network structure. We addressed this question with a sensitivity analysis for two different applications that are inspired by recent experimental and theoretical studies: the quantification of scale-free properties of the network and the identification of hub neurons with imperfect network reconstruction.

**Figure 7:**
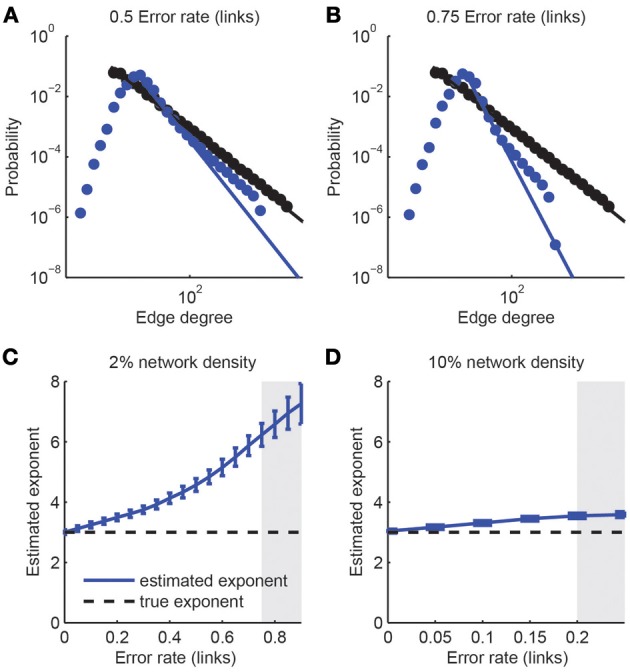
**Detection of scale-free graphs upon imperfect connectivity reconstruction. (A,B)** Degree distributions of networks after simulated reconstructions. The degree distribution of the original network (1000 neurons, 4% link density) follows a power law *p*(*x*) ~ *x*^−3^ above a minimal degree (black dots and line). Reconstructed networks (blue dots) were obtained with varying error rate in the link reconstruction, ranging from 0.50 (left) to 0.75 (right). In all cases the best-fitting power law to the tail of the degree distribution is indicated (blue line). **(C,D)** Estimated power-law coefficients, obtained from power-law fits to the tails of estimated degree distribution, as a function of error rate in link reconstruction for different network densities. Error bars: standard deviation over 1000 simulations. The coefficient of the original network is indicated by the horizontal dashed line. A goodness-of-fit test was applied to each fit. If more than 50% of the *p*-values were below 0.05, the region is grayed out, indicating that the resulting degree distributions were generally inconsistent with the power-law assumption.

First, we considered the class of *scale-free networks* (Barabasi and Albert, 1999): Scale-free networks are characterized through a degree distribution that follows a power law *p*(*x*) ~ *x*^−μ^, where the probability that a neuron of the network has *x* synaptic connections scales like a power law with characteristic exponent μ. We generated prototypical examples of scale-free networks of size *N* = 1000 neurons and exponent μ = 3 and analyzed the impact of expected network reconstruction performance with varying error rates α_links_ and assuming that the identification of a link between any two neurons is statistically independent of any other connection (Figure 7). Thus, we obtained simulated reconstructed networks that differed in their statistical properties from the original scale-free network. We fitted power-law distributions to the reconstructed degree distributions and assessed goodness-of-fit with a semi-parametric bootstrapping method (Figures 7A,B). Although link over- and under-sampling does in general not result in a pure power-law (Han et al., 2005; Stumpf et al., 2005), many of the resulting networks were still compatible with the scale-free assumption for the tail of their degree distributions (*p*_gof_ > 0.05). However, the estimated exponent could be considerably different from the original one. In general, we found a stronger decay of the degree distribution (more negative power law exponent) upon imperfect network reconstruction (Figures 7C,D), regardless of the underlying network density (varied between 2 and 10%), i.e., the number of links present in the network. Only if reconstruction errors get moderately high (e.g., α_links_ > 0.75 for networks with 2% link density), the power-law distribution was not evident anymore. The difference between the fitted and original scaling exponent varied with the network density. For denser networks, the sensitivity to imperfections of the detection of the scale-free property based on the data sample is increased. For example, an error rate of up to 0.75 was tolerated for sparsely connected networks (density 2%) while an error rate of 0.2 (which is not realistically achievable under experimental conditions) was sufficient to make the degree distribution distinct from a power-law for denser networks (10% connectivity; Figure 7D). A detailed analysis of the contributions separate from missed links and false positive links indicated that the consistent over-estimation of the power-law exponent was mostly due to the introduction of false positive links (results not shown) while it remained almost unaffected by non-perfect TPR_links_. This suggests that when considering the trade-off between TPR_links_ and FDR_links_, emphasis should be put to minimize the number of false positive links on the expense of the link detection power.

**Figure 8:**
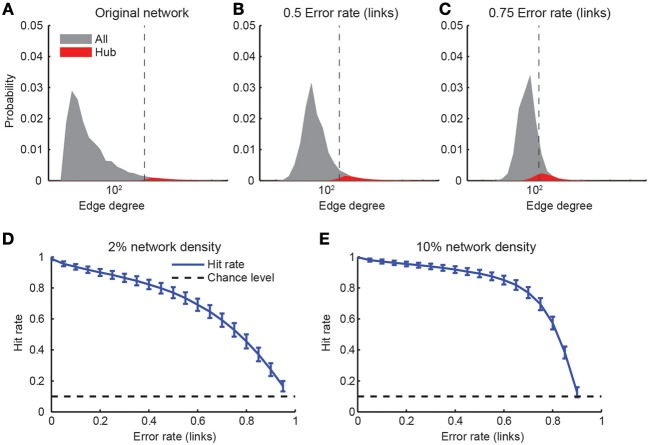
**Detection of hub neurons upon imperfect connectivity reconstruction. (A–C)** Degree distributions of networks after simulated reconstructions. The degree distribution of the original network **(A)** followed a power law *p*(*x*) ~ *x*^−3^ above a minimal degree of 20. Hub neurons were defined as the 10% of neurons with the highest degree (red areas). Imperfectly reconstructed networks were obtained by assuming varying error rates **(B,C)**. Vertical dashed lines indicate the beginning of the upper-most decile of the estimated distribution. **(D,E)** Hit rate of hub neuron identification as a function of varying degree of link reconstruction error for two different network densities. Error bars: standard deviation over 1000 simulations. Chance level indicated by dashed lines.

*Hub neurons* are another concept relevant for graph theory (Feldt et al., 2011): Hub neurons are those neurons with a comparatively high degree, i.e., large numbers of incoming or outgoing connections in the network. As before, we started with networks of size *N* = 1000 neurons and a scale-free topology. We classified individual neurons as hub neurons when their degree was in the upper-most decile of the degree distribution (Figure 8A). Imperfect network reconstruction, for example due to non-perfect link reconstruction or the insertion of false positive links, generally scattered the degree distribution and therefore the identity of hub neurons. Consequently, not all original hub neurons remained within the top decile of the estimated degree distribution (Figures 8B,C). We quantified the robustness with regard to reconstruction errors with the “hit rate,” i.e., the fraction of hub neurons of the original network that were correctly classified as hub neurons in the reconstructed network. As expected, the hit rate decreased steadily with increasing error rate in the link reconstruction (Figures 8D,E), with only slight dependence on the original network density. Surprisingly, however, hub classification could still be robustly achieved (75% hit rate) for relatively high error rates (0.6–0.75). We conclude that hubs can be detected in a relatively robust manner even in the presence of large link reconstruction errors.

## Discussion

In this study we analyzed inference of spike dynamics in neuronal networks, as well as inference of underlying structural properties, based on population fluorescence data as they are typically acquired during *in vivo* two-photon calcium imaging experiments. We established a simulation framework that—unlike experiments—allows comprehensive exploration of parameter spaces. Systematic parameter variation helps to explore the limits of what is currently achievable, identify critical parameters, motivate further methods improvements, and guide the experimenter in the optimization of their imaging conditions. Our results indicate that with state-of-the-art methods, especially high-speed two-photon calcium imaging, it is now feasible to reconstruct spike trains in populations of several tens of neurons with high precision. Remaining uncertainties in exact spike times lower, but do not preclude, retrieval of partial information about network connectivity and topological features. *In vivo* calcium imaging combined with the analysis tools described here thus promises to become a powerful method to analyze the functional organization of neuronal networks in the brain.

### Potential and limits of spike inference

In the first part of this study, we highlighted the utility of our simulation framework to reveal non-trivial relations among the key imaging parameters and the accuracy of spike inference. Our systematic analysis of spike train reconstruction (here exemplified for the peeling algorithm) provides a resource for experimentalists, from which the expected reconstruction performance for a given set of experimental conditions can be obtained (Figures 2–5). Similar analyses can easily be implemented for alternative spike reconstruction algorithms (Yaksi and Friedrich, 2006; Vogelstein et al., 2009, 2010) and other experimental conditions. Our analysis reveals a number of novel insights. First, we show that near-optimal spike train reconstruction (i.e., detecting nearly all spikes with negligible numbers of false positives) may be achieved at experimentally tractable noise levels (*SNR* ≥ 2) with surprisingly low sampling rates (20–30 Hz). Frame scanning with conventional mirror-based laser scanners typically is limited to 10–20 Hz for 50–100 lines, as the standard galvanometer can be driven at about 1 kHz at maximum. Nonetheless, faster acquisition rates (even >100 Hz) are possible with standard scanners using free line-scans on preselected neuronal subsets (Göbel and Helmchen, 2007; Nikolenko et al., 2007; Lillis et al., 2008; Rothschild et al., 2010), albeit usually at the expense of total dwell time per cell (and thus SNR). Alternatively, full-frame scanning up to 100 Hz has been achieved by fast scanning along one axis with a resonant galvanometer (4–12 kHz resonance frequencies) (Rochefort et al., 2009; Bonin et al., 2011). All these methods with the capability of scanning neuronal populations at greater than video rate (25 Hz) should enable high-quality spike inference.

Two trade-offs, however, need to be considered. Gaining speed in the range of 10–100 Hz will only help to improve spike inference if a sufficient SNR is maintained (Figure 2A). In addition, effective sampling rate usually relates inversely to the number of recorded cells for a given SNR, so that a compromise between speed and population size is required. For a fair comparison of imaging approaches and spike reconstruction accuracies one should thus rely on populations of similar size. Fundamentally, these trade-offs arise from the fact that SNR is ultimately limited by photon statistics (Wilt et al., 2013). Of course, the SNR will be lower at higher frame rates given the same excitation power. However, high-speed scanning at low excitation rate can have the additional benefit of reduced phototoxicity and thus prolonged experiment time (Chen et al., 2012). In addition, detection of fluorescence photons should be maximized, for example by using a low-magnification, high numerical aperture objective for detection (Oheim et al., 2001) or by employing supplementary detection schemes (Engelbrecht et al., 2009).

A second insight is that ultra-fast imaging (sampling rates >500 Hz) in combination with high SNR levels permits spike reconstruction with millisecond or even sub-millisecond precision. Such high acquisition rates for neuronal populations, e.g., 0.5 kHz for about 50 neurons, are possible with random-access scanning using acousto-optical deflectors (Reddy and Saggau, 2005; Grewe et al., 2010; Ranganathan and Koester, 2010). To fully exploit the potential of highest-speed calcium imaging it will be essential, however, to reduce additional noise sources such as baseline fluctuations, bleaching effects, or motion artifacts to a minimum.

Our analysis furthermore suggests that the combination of ultra-fast imaging and high SNR may be achievable with the next generation of highly sensitive GECIs (Horikawa et al., 2010; Chen et al., 2013b). Our results reveal that faster decay kinetics of the indicator does not necessarily lead to better spike reconstruction. Intuitively, if the decay time of the calcium indicator dye is faster than the frame duration, peaks will occasionally be missed, thereby reducing detection accuracy. Thus, emerging faster calcium indicators, especially new GECIs, might be of limited use unless combined with new microscopy techniques that allow for faster image acquisition. In addition, because slow onset kinetics slightly reduces the achievable temporal precision, GECIs with fast onset characteristics are desirable if uncertainty of spike times needs to be minimized, for example to extract network structural properties.

### Inferring network structure from calcium imaging data

Our simulation framework summarizes the effects of noise, calcium indicator dynamics, and imperfect reconstruction with two key quantities: the error rate (a combination of the fraction of correctly identified spikes and the rate of erroneously detected spikes) and the precision of reconstructed spike times. Based on these parameters, we could estimate how well we are likely to be able to estimate network connectivity. The goodness of connectivity estimation may again be summarized by the same key metric: the error rate in the network link reconstruction. This parameter has an influence on the estimation of graph properties, as we have exemplified for scale-free topology and the identification of hub neurons. Similarly, our framework should allow analysis of the robustness of other graph statistics such as small-world properties or the distribution of higher-order network motifs.

The modular structure of our framework enabled us to predict the effect of experimental parameters on the global estimation of statistical graph properties by simply following its effect through the different stages. For the benefit of experimenters, we can illustrate the utility of our simulation framework with a practical example, assuming state-of-the-art technology. Using the high-affinity calcium indicator OGB-1 in combination with AOD-based random-access sampling at ≈500 Hz, neuronal calcium signals have been measured in mouse neocortex with SNR up to 5 (Grewe et al., 2010). These parameters will allow spike reconstruction with near-perfect accuracy (Figure 3A, *TPR*_AP_ > 0.95, *FDR*_AP_ < 0.05, therefore, α_AP_<0.05) as well as 2–3 ms temporal precision (Figure 5C, inset). In this parameter range, spike detection is sufficiently accurate so that no information loss is expected to occur with respect to the reconstruction of synaptic connections (Figure 6E, left). The imperfect temporal precision, however, leads to a slightly increased error rate in the link reconstruction compared to sub-millisecond temporal precision (Figure 6E, right). The approximate error rate can be also directly read from Figure 6F. Under otherwise optimal conditions, we would obtain an error rate α_links_ ≈ 0.60, therefore we expect to recover around 40% of the synaptic connections (TPR_links_ = 0.4) with FDR_links_ = 0.6. According to this estimate, robust identification of hub neurons should still be feasible (Figures 8D,E) and scale-free properties might be just identifiable (but heavily biased) depending on the underlying connection density (Figures 7C,D).

The absolute numbers in the example above should be regarded as ballpark estimates rather than precise predictions of reconstruction performance because inference is based purely on simulated data and our particular choice of algorithms. Real reconstruction performance could be weaker than predicted due to effects such as unobserved neuromodulation, weak synaptic strengths, or oscillatory background activity. In addition, connectivity reconstruction could potentially be improved by using more complex point process models that explicitly model global state fluctuations (Smith and Brown, 2003) by attempting to infer the dynamics of unobserved neurons (Vidne et al., 2012) or by employing Bayesian methods (see below).

Few other studies explored the possibility of reconstructing network connectivity from calcium imaging data. Mishchenko and colleagues presented a sensitivity analysis using a Bayesian approach combined with MCMC (Markov Chain Monte Carlo) techniques (Mishchenko et al., 2011). The combined estimation of spike times and connectivity make their approach computationally very expensive. Our modular approach offers the advantage that we can identify the crucial experimental parameters and propagate their effect through the spike time estimation to the network level. Mishchenko et al. did not consider the effect of imperfect link reconstruction for the inference of higher-level topological features of the network. Our results suggest that the optimal choice of experimental parameters can strongly depend on which feature of the network one wants to estimate most reliably. Finally, we note that their recommendation to use frame rates of at least 30 Hz “to achieve meaningful reconstruction results” (Mishchenko et al., 2011) is in alignment with our findings, although both methodology and details of the simulation vary considerably between approaches.

Another study proposed a method based on information-theoretic measures to infer effective connectivity from calcium imaging experiments and evaluate it on simulated data (Stetter et al., 2012). Our approach extends their analysis in a number of different aspects. First, their approach is only suitable for recordings with low sampling frequencies. The information-theoretic measure (transfer entropy) they use to infer couplings does not easily scale up to high-speed recordings because of the need to estimate high-dimensional probability distributions. Activity levels were coarsely discretized, which will have a negative impact on the performance especially for low SNRs. Second, their measure of coupling strength cannot distinguish between excitatory and inhibitory couplings, is pairwise only (i.e., it does not take into account the activity of other recorded cells) and is limited in how temporal aspects of couplings between cells can be modeled. All of these limitations can be overcome by the use of non-linear point process models based on the estimated spike trains, as proposed in this study. Last, (Stetter et al., 2012) did not study the impact of different experimental conditions and can therefore give only limited guidance on how to design experimental set-ups.

We note that we use the peeling algorithm coupled with point process models as an example of a combination of methods to extract connectivity. We favored a two-step procedure of first reconstructing spike trains and then inferring connectivity based on estimated spike times over directly modeling couplings between fluorescence traces (Stetter et al., 2012; Turaga et al., in press). Given that spikes are triggering neurotransmitter release at synapses (at least for most cortical cell types) we expect our approach to be closer to the biological mechanism of how neural signals are coupled, and therefore to be superior in estimating connectivity. A formal comparison would though require the evaluation of both methods on the same simulated data sets.

Due to our modular analysis, the last part of our study (how high-level network properties can be recovered given an expected link reconstruction performance) is independent of the underlying methods to infer connectivity once the performance of any such reconstruction method is quantified in terms of expected link detection power and false discovery rates. Thus, a similar sensitivity analysis could be performed for additional network measures (such as small-world properties or network motifs), and our conclusions readily apply to connectivity estimates obtained from electrophysiology, e.g., from multi-electrode arrays or other imaging modalities.

### Future directions

Two-photon calcium imaging has conventionally been calibrated by simultaneous imaging and electrophysiological recording of single neurons (Kerr et al., 2005). Based on the ground truth provided by the electrical recording, the performance of spike inference from calcium imaging data can be verified, albeit the extent of such analysis (e.g., to test various experimental conditions) is limited due to the technical difficulties. Similarly, connectivity inference and extraction of topological network properties will eventually require experimental verification against ground truth data. A first attempt has been made by Gerhard et al. (2013) who showed that the effective connectivity derived from spiking activity using a point process model similar to the one used here matches the physiological connectivity in a very small, but well-defined neural circuit. While it remains a difficult task to test these methods on larger populations, novel approaches have recently emerged that at least partially may allow such verification, including large-scale anatomical circuit reconstructions using electron microscopy (Bock et al., 2011; Briggman et al., 2011) and automated light-microscope techniques in combination with expression of cell type–specific markers and trans-synaptic tracers (Osten and Margrie, 2013). In addition, connectivity mapping can be performed following *in vivo* calcium imaging and re-identification of the recorded neuronal populations in extracted tissue using various physiological techniques, such as multi-cell electrophysiological recordings in acute brain slices (Hofer et al., 2011; Ko et al., 2011) or two-photon photo-stimulation with single-cell resolution using caged compounds or specially tailored opsins (Prakash et al., 2012). At present, these methods are, however, limited to specific individual neurons or small groups of neurons at most.

Our study may be extended in several directions. In particular, the heterogeneity of neuronal cell types could be taken into account. All above considerations straightforwardly apply to superficial neocortical pyramidal neurons, which produce large single-AP evoked calcium transients and display relatively low spontaneous firing rates. Our extension of the peeling algorithm to account for indicator saturation should also allow reconstruction of brief AP bursts and episodes of higher firing rates, which is especially relevant for awake studies (Greenberg et al., 2008; Wolfe et al., 2010) and for deep-layer cortical pyramidal neurons that generally display higher AP rates (De Kock and Sakmann, 2008). Inhibitory interneurons, especially fast-spiking parvalbumin-expressing cells, have much smaller single-AP evoked calcium transients as well as higher firing rates (Hofer et al., 2011), suggesting that accurate spike inference may not be feasible for these neurons (*SNR* presumably below 0.5). On the other hand, recent *in vitro* electrophysiological work indicated that inhibitory neurons form a relatively unspecific and densely connected network in neocortical circuits (Fino and Yuste, 2011; Packer and Yuste, 2011). Another promising direction of future calcium imaging studies is to resolve the precise functional and structural topology of highly specific local networks of pyramidal neurons (Song et al., 2005). Our findings indicate that the technical requirements to achieve this goal may be just in reach.

## Materials and methods

### Simulation of single-neuron spike trains, calcium dynamics, and indicator fluorescence signals

Simulation of single-neuron spike trains and calcium indicator fluorescence signals and all analysis were performed in Matlab (The Mathworks, Natick, MA, USA). We generated spike trains by a Poisson process assuming a low mean firing rate (0.2 Hz) similar to what has been reported for spontaneous activity of pyramidal cells in both anesthetized and awake rodent sensory cortex (Wolfe et al., 2010). In addition, to examine the effect of calcium indicator saturation we explored episodes of higher firing rates between 1 and 30 Hz as they occur in pyramidal neurons, e.g., upon sensory stimulation (Greenberg et al., 2008).

A general description of AP-evoked fluorescence signals needs to consider the transformation of changes in intracellular free calcium concentration [Ca^2+^]_*i*_ to the particular type of fluorescence readout. Here, we use the widely adopted Δ*F*/*F* approach, expressing calcium signals as relative percentage fluorescence changes after background subtraction. In this case the transformation between [Ca^2+^]_*i*_ and fluorescence signal is given by (Helmchen, 2012):

(1) $${\left[{\text{Ca}}^{2+}\right]}_{i}=\frac{{\left[{\text{Ca}}^{2+}\right]}_{\text{rest}}+K_{d}\frac{\Delta F/F}{\Delta F/F_{\text{max}}}}{\left(1-\frac{\Delta F/F}{\Delta F/F_{\text{max}}}\right)}$$

or reversely expressed by:

(2) $$\Delta F/F=\Delta F/F_{\text{max}}\frac{{\left[{\text{Ca}}^{2+}\right]}_{i}-{\left[{\text{Ca}}^{2+}\right]}_{\text{rest}}}{{\left[{\text{Ca}}^{2+}\right]}_{i}+K_{d}}$$

Here, [Ca^2+^]_rest_ denotes the resting calcium concentration, *K*_*d*_ the dissociation constant of the calcium indicator, and Δ*F*/*F*_max_ the maximal Δ*F*/*F* reached upon saturation. Note that this transformation is a non-linear relationship. For fluorescence transients far from saturation ([Ca^2+^]_*i*_ « *K*_*d*_) Equation 2 can be linearized to:

(3) $$\Delta F/F\text{​}=\text{​}\frac{\Delta F/F_{\text{max}}}{K_{d}}({\left[{\text{Ca}}^{2+}\right]}_{i}-{\left[{\text{Ca}}^{2+}\right]}_{\text{rest}})=\frac{\Delta F/F_{\text{max}}}{K_{d}}\Delta {\left[{\text{Ca}}^{2+}\right]}_{i}$$

**Figure 9:**
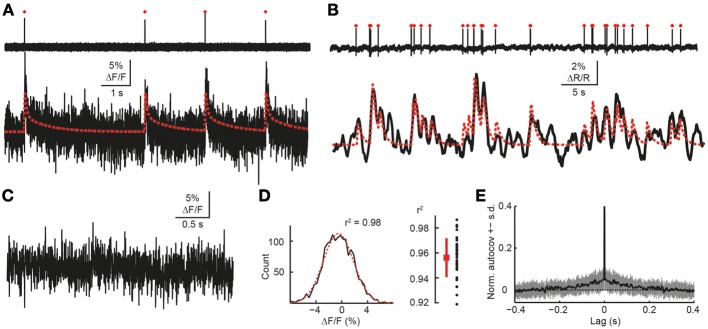
**Validation of simulation framework with experimental data. (A)** Simultaneous cell-attached recording and high-speed two-photon calcium imaging in mouse neocortex *in vivo*. Top trace: cell-attached recording, APs marked by red dots. Bottom trace: measured cellular Δ*F*/*F* calcium signal (black) as well as simulated Δ*F*/*F* traces (red) for the recorded spike train. Imaging data were acquired with OGB-1 at 490 Hz sampling rate (Grewe et al., 2010). Note that a non-saturating model with double-exponential decay was used in this case to generate the simulated trace. **(B)** Simultaneous cell-attached recording and two-photon calcium imaging using the genetically-encoded calcium indicator YC3.60 (Lütcke et al., 2010). Top trace: cell-attached recording, APs marked by red dots. Bottom trace: measured cellular calcium signal (black) as well as simulated Δ*F*/*F* traces (red) for the recorded spike train (sampling rate: 7.81 Hz; expressed as relative percentage change Δ*R*/*R* of the YFP/CFP fluorescence ratio). A non-saturating model with single-exponential decay was used to generate the simulated trace. **(C)** Noise Δ*F*/*F* trace during episode without AP, as confirmed by simultaneous cell-attached recording (data not shown). Sampling rate: 490 Hz. **(D)** Left: distribution of signal intensities for data shown in **(C)**. Gaussian fit in red (*r*^2^ = 0.98). Right: distribution of goodness-of-fit of Gaussian fits (*r*^2^) for pooled data set (96 s total recording time). **(E)** Mean normalized autocovariance (±SD) for pooled noise data set. Peak at 0 s lag clipped.

This linear description is a good approximation for AP-evoked fluorescence signals in the low firing regime measured for example with a high-affinity indicator such as OGB-1 (Grewe et al., 2010) (Figures 9A,B). In this case each AP evokes a stereotype, elementary somatic calcium transient, which can be approximated with a rapidly rising and exponentially decaying function:

(4) $$\Delta F/F=A\left(1-e^{-\left(t-t_{0}\right)/\tau_{\text{on}}}\right)e^{-\left(t-t_{0}\right)/\tau_{\text{off}}}\text{ for }t\geq t_{0}$$

Here, *t*_0_ denotes the time point of spike occurrence, τ_on_ the onset rise time, τ_off_ the decay time, and *A* an amplitude scale parameter. The peak amplitude *A*_Peak_ of the single-AP evoked calcium transient is given by:

(5) $$A_{\text{peak}}=A\tau_{\text{off}}{\left(\frac{\tau_{\text{on}}}{\tau_{\text{on}}+\tau_{\text{off}}}\right)}^{\frac{\tau_{\text{on}}}{\tau_{\text{off}}}}{\left(\tau_{\text{on}}+\tau_{\text{off}}\right)}^{-1}$$

For the calcium indicator OGB-1, typical values of these parameters for neocortical pyramidal neurons are τ_on_ = 10 ms, *A*_peak_ = 7% Δ*F*/*F*, τ_off_ = 0.5–1 s (Grewe et al., 2010). For the low firing regime we used the canonical elementary Δ*F*/*F* transient (Equation 4) as impulse response function. Other more complex shapes of the elementary transient, for example a double-exponential decay (Grewe et al., 2010), could be easily incorporated into the simulation framework. Because of the linear approximation, we obtained the fluorescence traces for the entire duration of the simulations by convolving the simulated spike trains with this elementary Δ*F*/*F* transient.

At higher AP firing rates, [Ca^2+^]_*i*_ may reach levels sufficiently high to cause substantial saturation of the calcium indicator. We therefore incorporated the possibility to account for indicator saturation in our simulation framework. Assuming a non-cooperative calcium binding characteristics, the saturation level *S* (ranging from 0 to 1) is given by:

(6) $$S=\frac{\left[\text{CaB}\right]}{{\left[\text{B}\right]}_{T}}=\frac{{\left[{\text{Ca}}^{2+}\right]}_{i}}{{\left[{\text{Ca}}^{2+}\right]}_{i}+K_{d}}=\frac{{\left[{\text{Ca}}^{2+}\right]}_{\text{rest}}+K_{d}\frac{\Delta F/F}{\Delta F/F_{\text{max}}}}{{\left[{\text{Ca}}^{2+}\right]}_{\text{rest}}+K_{d}}$$

Here, [B]**_*T*_ denotes the indicator concentration in the cell and the equation's right side was obtained by insertion of equation 1. Importantly, indicator saturation not only leads to a non-linear transformation between [Ca^2+^]_*i*_ and Δ*F*/*F* but also directly affects buffered [Ca^2+^]_*i*_ dynamics, an aspect that has been neglected in previous attempts to incorporate indicator saturation in spike inference algorithms (Vogelstein et al., 2009; Stetter et al., 2012). Differentiation of Equation 6 with respect to [Ca^2+^]_*i*_ yields the so-called Ca^2+^-binding ratio κ_B_ (or “buffering capacity”) of the indicator, which decreases with increasing [Ca^2+^]_*i*_ levels near saturation:

(7) $$\kappa_{B}={\left[\text{B}\right]}_{T}\frac{\partial S}{\partial {\left[{\text{Ca}}^{2+}\right]}_{i}}=\frac{\partial [\text{CaB}]}{\partial {\left[{\text{Ca}}^{2+}\right]}_{i}}=\frac{{\left[\text{B}\right]}_{T}K_{d}}{{\left({\left[{\text{Ca}}^{2+}\right]}_{i}+K_{d}\right)}^{2}}$$

Note that the Ca^2+^-binding ratio critically depends on the indicator's Ca^2+^-binding affinity and its total concentration. The effect of adding an exogenous Ca^2+^-buffer such as the indicator on AP-evoked somatic calcium signals is well-understood for neocortical pyramidal neurons and is typically approximated by a single-compartment model, which assumes chemical equilibrium and neglects diffusion (Helmchen and Tank, 2011). The model additionally considers an endogenous Ca^2+^-binding ratio κ_S_, which we assumed to be constant [κ_S_ = 100; (Helmchen et al., 1996)], and the Ca^2+^ extrusion rate γ (800 s^−1^) (Helmchen and Tank, 2011). [Ca^2+^]_rest_ was assumed 50 nM. The relaxation of [Ca^2+^]_*i*_ from an elevated level back to resting level is then described by the following non-linear differential equation:

(8) $$\begin{array}{l} \frac{d{\left[{\text{Ca}}^{2+}\right]}_{i}}{dt}\text{​}=\text{​}\frac{-\gamma \;\Delta {\left[{\text{Ca}}^{2+}\right]}_{i}}{\left(1+\kappa_{S}+\kappa_{B}\right)}\\ \text{ ​}=\text{​}-\gamma \text{​}{\left(\text{​}1+\kappa_{S}+\frac{{\left[\text{B}\right]}_{T}K_{d}}{{\left({\left[{\text{Ca}}^{2+}\right]}_{i}+K_{d}\right)}^{2}}\text{​}\right)}^{-1}\text{​​}\left({\left[{\text{Ca}}^{2+}\right]}_{i}-{\left[{\text{Ca}}^{2+}\right]}_{\text{rest}}\right) \end{array}$$

To calculate the model [Ca^2+^]_*i*_ traces for a given spike train, we numerically solved Equation 8 for each spike-to-spike interval starting from the [Ca^2+^]_*i*_ level reached after each AP. This level was calculated by incrementing the pre-AP [Ca^2+^]_*i*_ level at the moment of the next spike's occurrence *t*_spike_ by

(9) $$\begin{array}{l} \Delta {\left[{\text{Ca}}^{2+}\right]}_{i}(t_{\text{spike}})=\frac{\Delta {\left[{\text{Ca}}^{2+}\right]}_{T}}{\left(1+\kappa_{S}+\kappa_{B}\right)}\\ \;=\text{​}{\left(\text{​​}1+\kappa_{S}+\frac{{\left[\text{B}\right]}_{T}K_{d}}{{\left({\left[{\text{Ca}}^{2+}\right]}_{i}(t_{\text{spike}})+K_{d}\right)}^{2}}\text{​​}\right)}^{-1}\Delta {\left[{\text{Ca}}^{2+}\right]}_{T} \end{array}$$

Here, Δ[Ca^2+^]_*T*_ denotes the total intracellular calcium concentration change caused by an AP, which was assumed 7.6 μM. The reduction of κ_B_ at elevated [Ca^2+^]_*i*_ levels due to indicator saturation thus leads to an increase of Δ[Ca^2+^]_*i*_ per AP. The sharp increments of [Ca^2+^]_*i*_ for each spike were smoothed with an exponential rising onset function (τ_on_ = 20 ms) similar to Equation 4. Finally, we transformed the [Ca^2+^]_*i*_ trace to a Δ*F*/*F* trace using equation 1, presuming the following reasonable parameter values for OGB-1: *K*_*d*_ = 250 nM, [B]_*T*_ = 50 μM, Δ*F*/*F*_max_ = 93%. With these parameter settings, a single-AP evoked Δ*F*/*F* transient from resting [Ca^2+^]_*i*_ level was similar to the stereotype Δ*F*/*F* transient described by Equation 4. Note that despite the increased Δ[Ca^2+^]_*i*_ at elevated [Ca^2+^]_*i*_ levels the non-linear transformation between [Ca^2+^]_*i*_ and Δ*F*/*F* (Equation 1) has the effect that the Δ*F*/*F*-increment per AP becomes small closer to saturation (see Figure 4B).

For both the linear (low firing rate) and non-linear (higher firing rates) case, we added Gaussian white noise with standard deviation SD_noise_ to the simulated Δ*F*/*F* traces. We assumed a realistic range of signal-to-noise ratios (SNR) for AP-evoked calcium transients, where we defined SNR as:

(10) $$\text{SNR}=\frac{A_{\text{peak}}}{SD_{\text{noise}}}$$

We verified the assumption of Gaussian noise by empirically determining the noise distribution from random-access calcium imaging data (OGB-1; 490 Hz scan rate) (Grewe et al., 2010) when no spike had occurred (as verified by simultaneous electrophysiology). Without exception, noise distributions could be well-approximated by fitting a Gaussian curve (*r*^2^ = 0.96 ± 0.02), suggesting that residual noise in two-photon calcium imaging indeed can be assumed normally distributed (Figures 9C,D) and contains little, if any, auto-correlation at lags >0.1 s (Figure 9E). Gaussian noise is a reasonable assumption because the number of detected photons is likely to be much greater than 100 under two-photon imaging conditions (Ranganathan and Koester, 2010). We note that for extremely low light conditions this assumption may not be valid. As the last step in our generation of simulated Δ*F*/*F* traces, we subsampled the resulting noisy Δ*F*/*F* trace from the original temporal resolution of 2 kHz to a given target frame rate, *f*, by selecting the center data point for each time interval Δ*t*, where Δ*t* = 1/*f*.

In summary, our analysis indicates that the presented simulation framework provides a valid model for AP-evoked calcium signals measured *in vivo* using two-photon microscopy. While experimental data may be characterized by additional noise sources not captured in our model (for example slow drifts or motion artifacts), these are generally easy to identify and remove prior to further data analysis. Whereas the linear description is appropriate for many cases and has been widely adopted (Yaksi and Friedrich, 2006; Vogelstein et al., 2010; Mishchenko et al., 2011), we have here also generalized our approach to the non-linear regime by considering indicator saturation. Extension to include further non-linearities—such as for example saturation of endogenous buffers, cooperative indicator Ca^2+^-binding, e.g., for GECIs (Pologruto et al., 2004; Horikawa et al., 2010; Chen et al., 2013b), or diffusional equilibration—will be straight forward. Likewise, other non-linear relationships between [Ca^2+^]_*i*_ and fluorescence readouts different from Δ*F*/*F*, for example using ratiometric measurements, could also be considered.

### Reconstruction of spike trains from calcium indicator signals

Action potentials were recovered from simulated Δ*F*/*F* traces using the peeling algorithm that we have introduced previously (Grewe et al., 2010). Briefly, AP-evoked fluorescence signal events were detected using Schmitt-trigger thresholding (high threshold: +1.75 SD, low threshold: −1 SD, minimal duration: 0.3 s) with additional integral check (at least 50% of theoretical noise-free integral). In the original peeling algorithm we assumed a linear relationship between [Ca^2+^]_*i*_ and Δ*F*/*F*, which we also applied here for the low firing regime. Specifically, a stereotype single-AP evoked Δ*F*/*F* transient waveform (with the same parameters as used for the simulation of [Ca^2+^]_*i*_ transients, unless noted otherwise) was iteratively subtracted (“peeled off”) as long as the integral of the residual trace remained positive and threshold-passing occurred.

An advantage of the model-based nature of the peeling algorithm is that a non-linearity like indicator saturation can be easily incorporated. Here, we extended the peeling algorithm to take saturation into account, in order to enable spike reconstruction from saturating Δ*F*/*F* traces at high AP firing rates (up to 30 Hz). To this end, the single-AP evoked Δ*F*/*F* transient was re-calculated for each AP taking the respective pre-AP [Ca^2+^]_*i*_ level into account (again presuming parameter values for OGB-1; see above). More specific, the [Ca^2+^]_*i*_-level dependent Δ*F*/*F* transient was calculated by taking the difference between the Δ*F*/*F* relaxation traces from post-AP and pre-AP levels (both computed by transforming the respective [Ca^2+^]_*i*_ decays, obtained by solving the differential Equation 8). For comparison of error rates we applied either the simple linear or the saturating peeling algorithm to [Ca^2+^]_*i*_ traces generated with a saturating indicator.

For both the linear and saturating peeling approach, the temporal precision of detected spikes was further improved by optimization of spike times (±1 s around the spike time determined with the peeling algorithm; ±0.1 s for high AP rates). Optimization was performed by minimizing the squared sum of the residual trace using a pattern search algorithm (implemented in the Matlab Optimization toolbox).

To examine spike detection performance independent of the particular Schmitt-trigger thresholds, we performed “precision-recall” (PR) analysis (see Table 1) by selecting combinations of Schmitt-trigger thresholds over wide ranges (high threshold: −2 to +5 SD; low threshold: −5 to +2 SD; minimal duration: 0–1 s) (Figures 2D,E). Within the framework of PR analysis (Davis and Goadrich, 2006), we defined the break-even point as the data point closest to the unity line. Error rate α_AP_ was defined as max(FDR, 1-TPR) at this point (range 0–1). Intuitively, α_AP_ describes the distance of the break-even point from the upper-right corner of the PR-curve, which represents optimal performance (Davis and Goadrich, 2006).

**Table 1:**
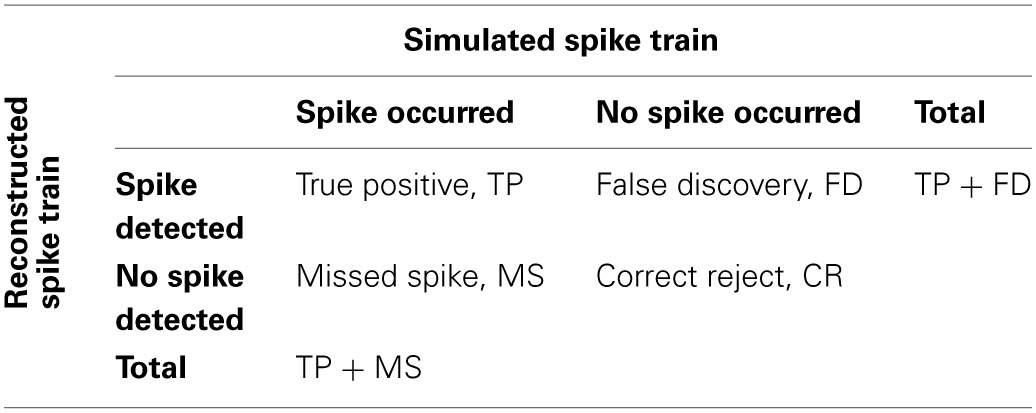
**Overview of spike metrics to quantify spike reconstruction accuracy**.

Simulated spikes may be either detected (true positive, TP) or missed (MS) by the reconstruction algorithm. We call the ratio of true discoveries to the total number of simulated spikes the True Positive Rate, TPR = TP/(TP + MS) or “recall.” On the other hand, spikes detected by the reconstruction algorithm may be matched by a simulated spike (true positive, TP) or represent false detections (false discovery, FD). We call the ratio of false discoveries to the total number of detected spikes the False Discovery Rate, FDR = FD/(TP + FD). In information retrieval theory (Davis and Goadrich, 2006), (1 − FDR) is also known as “precision.” Both TPR and FDR are defined as 0 for the special case of no simulated or reconstructed spikes, respectively.

### Comparison of original and reconstructed spike trains

Spike time comparison was performed by successively matching spikes in the original and reconstructed spike train based on ascending spike time difference (up to a maximal difference of 0.5 s, see Figure 1D). Remaining spikes in the original spike train reduce the true positive rate (calculated as fraction of total spikes in the original spike train) while spikes remaining in the reconstructed train contribute to the false discovery rate (calculated as a fraction of total spikes in the reconstructed spike train). We quantify reconstruction performance by the following parameters (Table 1):

1. True positive rate (TPR): fraction of correctly detected spikes (out of total spikes in original spike train); *TPR* ∈ [0, 1],
2. False discovery rate (FDR): fraction of false discoveries (out of total spikes in reconstructed spike train); *FDR* ∈ [0, 1],
3. Temporal precision: mean and standard deviation of spike time differences between original and reconstructed spike trains, meanΔ_*t*_ and σ_Δ*t*_, respectively (only for correct detections).

### Large-scale network simulation and detailed neuron model

We simulated a network of 25,000 leaky integrate-and-fire neurons with conductance-based synapses (Zenke et al., 2013). 80% of the neurons were modeled as excitatory and 20% as inhibitory. Connectivity was chosen randomly with a density of 10%. In addition, each neuron received common excitatory input from a pool of 2000 independent Poisson processes that were connected randomly to all neurons with 10% probability. The rate of the external input was modeled as a pink noise stochastic process with a mean firing rate of 2 Hz per process and exhibiting fluctuations on all time scales (1/*f* power spectrum) to mimick complex temporal dynamics of common-input in cortical networks. The network was tuned to the balanced state with asynchronous and irregular firing activity with a mean spiking activity of ~0.2 Hz.

Specifically, the membrane voltage *U*_*i*_ of a single cell *i* evolved according to:

(11) $$\tau^{m}\frac{dU_{i}}{dt}=\left(U^{\text{rest}}-U_{i}\right)+g_{i}^{\text{exc}}(t)\left(U^{\text{exc}}-U_{i}\right)+g_{i}^{\text{inh}}(t)\left(U^{\text{inh}}-U_{i}\right)$$

with membrane time constants τ^*m*^ = 20 ms for excitatory neurons and τ^*m*^ = 10 ms for inhibitory neurons, resting potential *U*^rest^=−70 mV, reversal potentials *U*^exc^ = 0 mV and *U*^inh^ = −80 mV and conductances *g*^exc^_*i*_(*t*) and *g*^inh^_*i*_(*t*) specified below. A spike was triggered when *U*_*i*_ crossed the spiking threshold ϑ_*i*_. After each spike, *U*_*i*_ was reset to the resting value *U*^rest^ and the threshold ϑ_*i*_ set to ϑ^spike^ = 50 mV to implement a refractoriness mechanism. Following a reset, the threshold exponentially decayed to its resting value ϑ^rest^ = −50 mV according to

(12) $$\tau^{\text{thr}}\frac{d\vartheta_{i}}{dt}=\left(\vartheta^{\text{rest}}-\vartheta_{i}\right)$$

with time constant τ^thr^ = 5 ms. The spike train *S*_*j*_(*t*) emitted by neuron j is given as *S*_*j*_(*t*) = ∑_*k*_ δ(*t* − *t*^*k*^_*j*_), where the sum runs over all k corresponding spike times *t*^*k*^_*j*_. Inhibitory synaptic conductances of the downstream neurons were affected by presynaptic spikes as:

(13) $$\tau^{\text{GABA}}\frac{dg_{i}^{\text{inh}}}{dt}=-g_{i}^{\text{inh}}+\sum_{j\;\in \text{ inh}}w_{ij}S_{j}(t)$$

with τ^GABA^ = 10 ms. Excitatory synapses were modeled containing a fast AMPA component with exponential decay (τ^AMPA^ = 5 ms) and a slow NMDA component (τ^NMDA^ = 100 ms):

(14) $$\tau^{\text{AMPA}}\frac{dg_{i}^{\text{AMPA}}}{dt}=-g_{i}^{\text{AMPA}}+\sum_{j\;\in \text{ exc}}w_{ij}S_{j}(t)$$

(15) $$\tau^{\text{NMDA}}\frac{dg_{i}^{\text{NMDA}}}{dt}=-g_{i}^{\text{NMDA}}+g_{i}^{\text{AMPA}}$$

The complete excitatory postsynaptic potential (EPSP) was obtained by a weighted sum of the AMPA and NMDA conductances:

(16) $$g_{i}^{\text{exc}}(t)=0.5g_{i}^{\text{AMPA}}(t)+0.5g_{i}^{\text{NMDA}}(t)$$

The weight values *w*_*ij*_ of the synapse connecting neuron *j* with *i* (*w*_*ij*_ = 0 if the connection does not exist) are given as follows: *w*(*E* → *E*) = *w*(*E* → *I*) = 0.2 and *w*(*I* → *E*) = *w*(*I* → *I*) = 0.9. The external Poisson inputs were connected with a constant weight *w*(ext → *E*, *I*) = 0.22. For computational efficiency, the voltage dependence of NMDA channels was omitted. All differential equations were integrated numerically using a forward Euler scheme with 0.1 ms time step using custom-written C/C++ code. Spike trains were generated for a total duration of *T* = 10,000 s.

### Connectivity reconstruction based on coupled point process models

We selected subsets of *N* = 50 excitatory neurons from the population that had an average firing rate of 0.6 Hz or higher and reconstructed the connectivity between neurons of this subpopulation based on their spike trains of length *T* = 10,000 s. To extract the coupling, we fitted coupled GLMs to the spike trains. Full details on the methodology can be found in (Gerhard et al., 2011). Briefly, spike trains are discretized into a sequence of binary values which represent spiking activity within time windows of length 1 ms. The instantaneous firing probability for each time bin is modeled as a non-linear transformation of the sum of covariates. These include effects from past spiking of the neuron itself as well as spikes from other neurons. All coupling filters are parameterized using a set of spline basis functions and parameters are estimated using standard maximum-likelihood techniques. Note that the strength of the stochastic common-input to each neuron is unobserved and therefore not explicitly modeled. The coefficients corresponding to the cross-coupling filters are used to define the effective coupling structure: The integral of each interaction filter represents its strength (Gerhard et al., 2013). A binary decision about the presence of a directed link can be enforced by thresholding the matrix of coupling strengths. The pair of TPR_links_ (fraction of correctly identified connections) and FDR_links_ (false discovery rate) defines the error rate for the link reconstruction as the smallest α_links_ that guarantees FDR_links_ ≤ α_links_ and TPR_links_ ≥ 1−α_links_. Results generally show the averaged performance derived from the analysis of several random subpopulations of the full network. To derive the expected error rate in the link reconstruction under the assumption that the effect of the absolute detection power (α_AP_) and spike time jitter (σ_Δ*t*_) act independently (Figure 6F), we use the intuition that detection powers ~1 − α would combine multiplicatively, so that, approximately:

(17) $$\alpha_{\text{links},\text{ independent}}\;\approx 1-\frac{\left(1-\alpha_{\text{links},\text{ due to }\alpha_{\text{AP}}}\right)\left(1-\alpha_{\text{links},\text{ due to }\sigma_{\Delta t}}\right)}{1-\alpha_{\text{links}}^{*}}$$

where α^*^_links_ is the best achievable error rate (in case of perfect spike reconstruction).

### Cross-correlation analysis

For comparison, we also implemented a connectivity extraction algorithm based on spike count correlations. We binned the spike trains into bins of size Δ*t*_cc_ and calculated the pairwise Pearson's cross-correlation coefficient of the resulting time series for each pair of neurons in the selected subpopulation. The negative logarithm of the significance value, i.e., the surprise, served as coupling strength. Note that this yielded symmetric (i.e., bidirectional) couplings. We swept through a wide range of values for Δt_cc_ (0.5–500 ms) and chose the one with best performance, resulting in Δ*t*_cc_ = 5 ms.

### Surrogate model of spike train reconstruction

We perturbed the spike trains using surrogate transformations to simulate the effect of the errors introduced by imperfect spike reconstruction from noisy calcium imaging data. Specifically, we used the two key parameters that were used to describe the performance of the single-neuron spike reconstruction (error rate α_AP_ and spike jitter σ_Δ*t*_). For any error rate α_AP_>0, spikes were randomly removed from the simulated spike trains to match the desired TPR_AP_. Simultaneously, spikes were added at random times up to the prescribed level of FDR_AP_. The temporal imprecision σ_Δ*t*_ was introduced by an additional jitter to all spike times given by a Gaussian distribution around zero with standard deviation σ_Δ*t*_. We repeated the connectivity estimation based on the perturbed spike trains and measured the performance using the error rate α_links_ whose value should be compared to the reference value achievable in the case of unperturbed original spike trains (assuming perfect spike time reconstruction).

### Identification of graph topology

#### Scale-free networks

We generated scale-free networks of size 1000 neurons by constructing unweighted, undirected graphs whose degree distributions follow a power law *p*(*x*) ~ *x*^−μ^ above a minimal degree *k* = 20 with exponent μ = 3, using the standard configuration model (Molloy and Reed, 1995). *k* was chosen as to produce an average link density of 4%, unless otherwise noted. We simulated the joint effect of calcium dynamics, spike train reconstruction and connectivity extraction by assuming that links are reconstructed with an error rate α_links_. This surrogate keeps the overall link density approximately constant. We then obtained the degree distribution of the reconstructed network and fitted a power law on its tail where the minimal degree and exponent were obtained using maximum-likelihood methods (Clauset et al., 2009). We constrained the exponent μ to be between 1 and 9 which covers all empirically observed scale-free networks. A goodness-of-fit test was applied to each fit using a Monte Carlo version of the Kolmogorov–Smirnov test (Clauset et al., 2009). We repeated the process of generation, imperfect reconstruction and re-fitting 1000 times and reported the median of the estimated power-law coefficient together with its standard deviation. We concluded that an estimated degree distribution was inconsistent with a power-law shape whenever the median *p*-value of the fit was below 0.05, i.e., a *p*-value < 0.05 occurred in more than half of the cases. Histograms of degree distributions were obtained with logarithmically spaced bins and by pooling distributions across all simulations.

#### Hub neurons

We generated scale-free networks of size 1000 neurons and power-law exponent μ = 3 as described above. We classified hub neurons in these networks as the 100 neurons with the highest degrees. We then simulated an imperfect network reconstruction as before and estimated how well neurons can be classified to be hub neurons as follows: The hit rate specifies the fraction of original hub neurons that belong to the 100 neurons with highest degree in the reconstructed network. A random assignment would lead to a hit rate of 10% (chance level). All estimates are based on 1000 simulations of independent networks.

### Conflict of interest statement

The authors declare that the research was conducted in the absence of any commercial or financial relationships that could be construed as a potential conflict of interest.
